# Supplementary material for: In Situ Pinpoint Photopolymerization of Phos-Tag Polyacrylamide Gel in Poly(dimethylsiloxane)/Glass Microchip for Specific Entrapment, Derivatization, and Separation of Phosphorylated Compounds
Source: Gels. 2021 Dec 16;7(4):268. doi: 10.3390/gels7040268 (PMC8701177; doi:10.3390/gels7040268)
Supplement: Supplementary file 1 [file gels-07-00268-s001.zip › gels-1449205-supplementary.pdf]

gels

Supporting Information

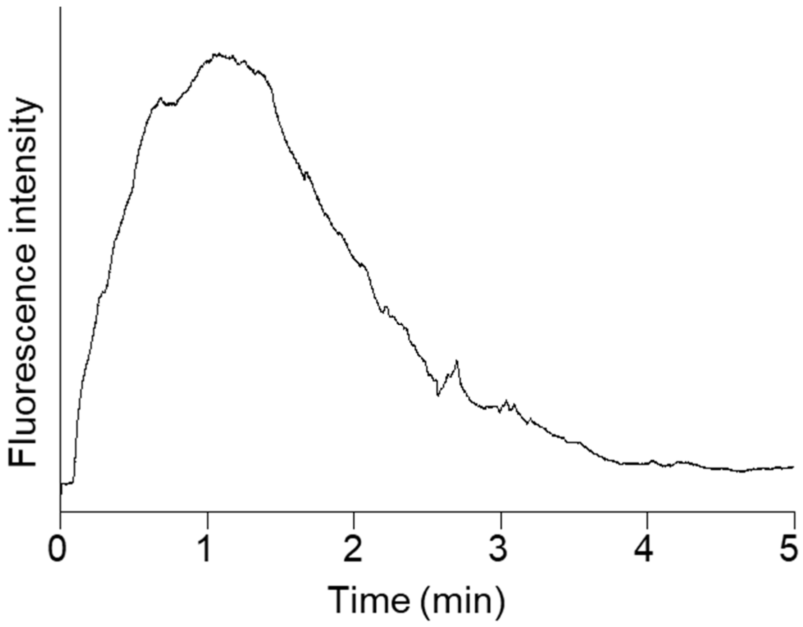

.

Figure S1. Time course of the changes in the fluorescence intensity due to introduction of 10^{-6} M DTAF at the Phos-tag acrylamide gel.

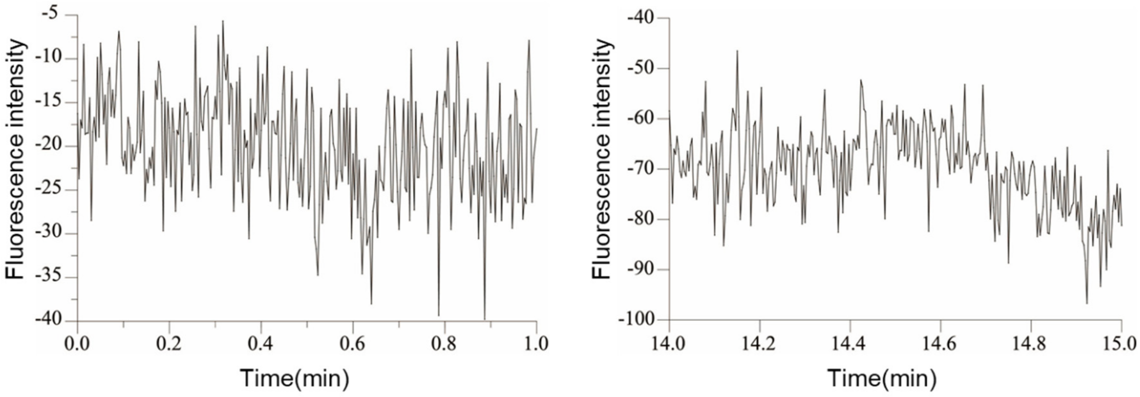

Figure S2. Time course of fluorescence intensity from 1 minute before the start of measurement and 1 minute before the end of measurement.

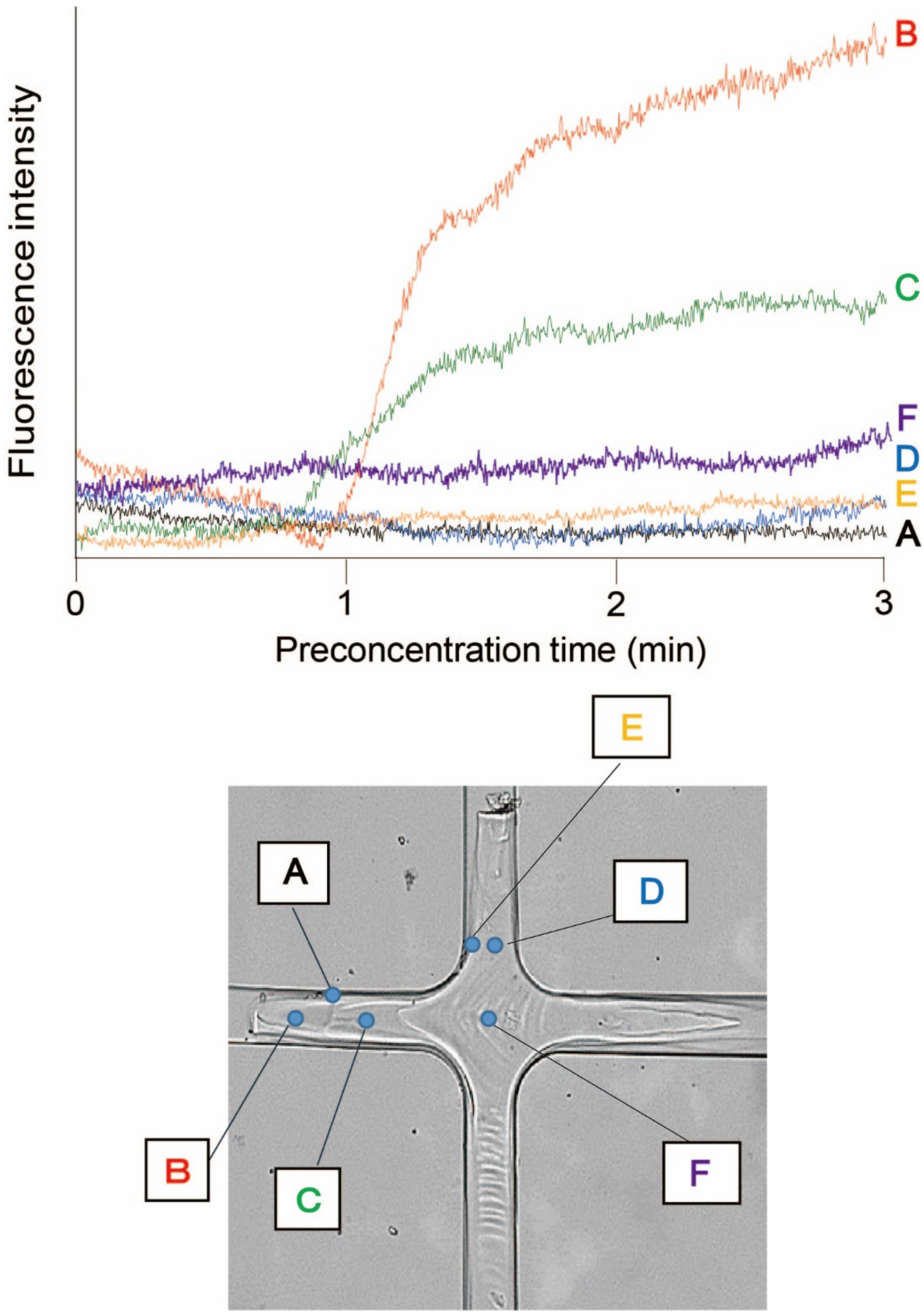

Figure S3. Time course of the changes in the fluorescence intensity due to concentration of the DTAF-labeled monophosphorylated β-casein at the various pin-point in the Phos-tag acrylamide gel. A-F shown in the figure below measures the fluorescence intensity of the corresponding part of the gel.
